# Predicting Molecular Subtypes of Breast Cancer with Mammography and Ultrasound Findings: Introduction of Sono-Mammometry Score

**DOI:** 10.1155/2021/6691958

**Published:** 2021-02-09

**Authors:** Sana Shaikh, Afshan Rasheed

**Affiliations:** ^1^Radiology Department, Sindh Institute of Urology and Transplantation (SIUT), Karachi 74100, Pakistan; ^2^Oncology Department, Sindh Institute of Urology and Transplantation (SIUT), M. A. Jinnah Road, Karachi 74100, Pakistan

## Abstract

We studied the correlation of sonographic and digital mammographic features with molecular classification of breast cancer. Imaging features from 313 patients with preliminary ultrasound and digital mammogram between November 2017 and May 2020 were compared with histopathology and immunohistochemical analysis for the prediction of molecular classification of breast cancer. We also devised a score called “sono-mammometry” score consisting of few simple imaging features which can easily be performed in outpatient settings. We studied that non-triple-negative breast cancers are predominantly hypoechoic and strongly correlate with the presence of irregular spiculated margins along with peripheral echogenic halo, posterior shadowing, and microcalcifications, while there is considerable variation in imaging features of TNBC as some of its imaging features overlap with those of typical benign tumors. Although imaging characteristics are helpful in the prediction of molecular classification, the prognostication value of these imaging features is still weak. There is considerable variation in imaging features which warrants vigilance towards improved diagnostic performance. To help better understand these features, our sono-mammometry score can serve as straightforward test which is assumed to be functional and productive in resource-limited settings.

## 1. Introduction

Breast cancer is the most common cancer in women and is the second most common cause of death from cancer in women worldwide [1]. It is a diverse group of diseases which consists of a wide range of molecular and genetic subtypes [2, 3]. During the last two decades, regrouping of breast cancer classification has been undertaken, from histopathologic subtype to the molecular categorization established by gene expression [1–4]. These subtypes exhibit significant differences in their clinical behavior and imaging pattern [2, 4]. These five different subtypes detected by immunohistochemical markers are luminal A, luminal B-1(Her-2−), B-2 (Her-2+), human epidermal growth factor receptor 2 (Her-2) enriched, and triple negative [1–5].

Breast imaging has a dominant role in the detection, staging, and follow-up of patients with breast cancer. There is a substantial effect on clinical outcome from early recognition of breast cancer [5]. Although histopathological characterization is the gold standard for classifying molecular subtypes of breast cancer after percutaneous breast mass biopsy, the trend is moving towards the development of noninvasive diagnostic procedures that can aid in easy and quick interpretation of the disease process [6]. This highlights the importance for a radiologist to have sound knowledge of these molecular subtypes of breast cancer to improve the analysis of imaging findings. The assessment of molecular subtypes of breast cancer on imaging perhaps displays intricacies as it requires the combination of imaging features on both ultrasound and mammography.

The purpose of our study is to identify the relationship of immunohistochemical markers with multimodality imaging features using mammogram and ultrasound. MRI breast was not available in our department. We also determined the association of baseline histopathological type, grade of tumor, and T and N stages of the breast cancers with each luminal classification. We also proposed a scoring system that is assumed to provide better insight into understanding imaging features of non-triple-negative breast cancer (non-TNBC) in contrast to triple-negative breast cancers (TNBC). Our hypothesis is that different sonographic and mammographic features can predict molecular classification on histopathology.

## 2. Materials and Methods

### 2.1. Study Population

Our study was conducted at the Radiology Department of Sindh Institute of Urology and Transplantation (SIUT). Our Institutional Ethics Committee waived the requirement of individual informed consent for this descriptive retrospective study. The study group was selected from both the screening and symptomatic populations. Patients with preliminary ultrasound, mammogram, and histopathological analysis done at our institution were regarded as being suitable for our study. Relevant information was gathered from ultrasound and mammogram images. Patients with incomplete records and unsatisfactory images and ductal carcinoma in situ (DCIS) were excluded from the study. Bilateral breast cancers were also excluded from the study because in our record we found only 5 such patients out of which in 3 patients we did not find satisfactory imaging. Very large inflammatory breast cancers were also excluded because these usually cause obscuration of fine details of ultrasound images of malignant masses secondary to excessive breast edema; however, locally advanced diseases in which breast edema was not present and we can comprehend ultrasound characteristics of malignant masses were included in the study. In this way, data from 313 patients with breast cancer were retrospectively collected between November 2017 and May 2020.

### 2.2. Ultrasound

All ultrasound examinations were done according to ACR practice parameters for the performance of a breast ultrasound examination (revised 2016) [7]. These were acquired according to the standard of care protocol as a routine practice of our breast cancer unit and these were not carried out under research study settings. For sonography, 2 scanners were used, both were Canon Xario 200, each with frequency of 14 MHz. Every sonographic examination included both breasts and was extended to involve both axillary regions.

### 2.3. Digital Mammography

All mammograms were performed according to ACR practice parameters for the performance of screening and diagnostic mammography (revised 2018) [8]. All mammograms were done with Selenia Dimensions 3D Digital Mammography Tomosynthesis System (Hologic, Bedford, MA, USA). Images were reviewed on high-resolution workstations at the Radiology Department of our institute. Standard 2 views (craniocaudal view and mediolateral view) were performed routinely for all patients. Supplementary views were done whenever required.

### 2.4. Ultrasound and Digital Mammogram Assessment

All ultrasound and mammogram images were reviewed by 1 of 2 senior consultants in breast imaging, both of whom had at least 5 years of experience in breast imaging. Both radiologists were blinded to the patient's clinical findings and histopathological results. In the case of disagreement, a consensus was reached after discussion. The sonographic and mammographic features of the breast carcinomas were assessed based on the analytical criteria of Breast Imaging Reporting and Data System (BIRADS). For ultrasound, these features include tumor size, shape, orientation, boundary, margins, echo texture, and posterior features. For the mammogram, the features that are assessed are mass shape, margins, presence or absence of suspicious microcalcifications, and architectural distortion/trabecular thickening. Additional features like T and N stages and multifocality were also assessed.

### 2.5. Histopathological Examination

The histopathological specimens from the core needle biopsy were formalin-fixed and paraffin-embedded tissue blocks afterwards stained with H&E (hematoxylin and eosin). These samples were assessed by an experienced pathologist for tumor type and grading. Invasive cancer was graded as grade 1 (well differentiated), grade 2 (moderately differentiated), or grade 3 (poorly differentiated) according to the Scarff–Bloom–Richardson system [9]. Subsequently, immunohistochemical analysis was done to assess ER, PR, Ki-67 index, and Her-2 neu expression. ER and PR were regarded as positive if at least 1% of the tumor nuclei were positively stained [10]. An additional fluorescence in situ hybridization (FISH) was analyzed to detect Her-2 positivity with scores of 2 or higher. Scores of 1 or 0 were defined as Her-2 negative [10]. Ki-67 index >14% was considered as high and <14% was considered as low expression [5].

### 2.6. Sono-Mammometry Score (Table 1)

**Figure 1:**
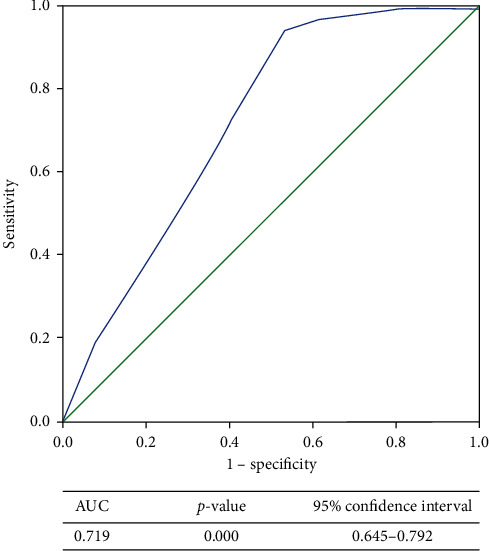
ROC curve.

We combined ultrasound and mammogram imaging features to design a scoring system for prediction of non-TNBC and TNBC. We used bivariate Chi-square test and multivariate binary logistic analysis to select variables. Variables with *p* values < 0.05 were considered significant. Factors significantly associated with univariable and multivariable logistic regression analysis are listed in Tables 2–4. Results of the univariable and multivariable logistic regression analysis were shown as *p* values, odds ratio (OR), and corresponding 95% CI. To estimate the discriminating power of the scoring system, the receiver operator characteristic (ROC) curve (Figure 1) was created and the areas under the curves (AUCs) were calculated.

By multivariate logistic regression analysis, seven variables on sonography and mammogram imaging were significantly related to predict non-TNBC from TNBC and were assigned scores for the final prediction. These are summarized in Table 5. These variables were as follows:

1. Presence of mass or focal asymmetry on mammogram or presence of mass on ultrasound: if not associated with microcalcifications (1 point); if associated with microcalcifications (2 points).
2. Mass shape on ultrasound or mammogram: round (1 point), oval (2 points), and if irregular (3 points).
3. Mass margins on ultrasound or mammogram: if well-circumscribed (1 point), microlobulated (2 points), and irregular-spiculated/ill-defined/obscured (3 points).
4. Mass boundary on ultrasound: abrupt (1 point); echogenic halo (2 points).
5. Posterior shadowing on ultrasound: absent (1 point); present (2 points).
6. Posterior enhancement on ultrasound: absent (1 point); present (1 point).
7. Orientation: parallel (+0); antiparallel (1 point).

This scoring system is not applicable in the absence of a sonographically and mammographically detected mass; therefore, score 0 was assigned to the presence of pleomorphic microcalcifications on mammogram without coexistent malignant mass on ultrasound and mammogram. Echogenicity was not included in the scoring system because in our study we did not find a significant difference among the echogenicity of non-TNBC and TNBC groups. The minimum and maximum scores were 6 and 14, respectively. The lower value of the score anticipates the presence of TNBC. In speculating TNBC, ROC curve analysis was performed for this sono-mammometry score, which showed significant statistical association (*p*=<0.01). AUC was demonstrated to be 0.719 with 95% CI of 0.645–0.792. At score 10.5, sensitivity is 94%, specificity is 47%, positive predictive value is 75%, and negative predictive value is 84%.

### 2.7. Statistical Analysis

The data was entered and analyzed in SPSS version 22.0. Mean and standard deviations were computed for continuous variables and categorical variables were presented as frequency and percentages and their comparison was done using bivariate Chi-square test. Multivariate binary logistic analysis was used to quantify the relative contribution of each imaging feature. *p* value <0.05 was considered as statistically significant. Odds ratios and confidence intervals were recorded for predictors of TNBC and Her-2 +ve disease. Receiver operating characteristic (ROC) analysis was performed and area under the curve (AUC) was recorded using the sono-mammometry scoring system devised for predicting imaging features for non-TNBC.

## 3. Results

### 3.1. Clinicopathological Findings (Table 2)

In our data set, the median age of patients was 49.5(+−12.4 S. D), out of which the majority of cancers were detected in 30–50 years of age. Among 313 patients, the most common histopathological type was infiltrating ductal carcinoma (*n* = 273, 87.2%), among which the most common was TNBC (*n* = 77, 24.6%) followed by luminal B1 (*n* = 71, 22.7%). Out of 10 (3.2%) metaplastic carcinoma (*n* = 7, 9.1%) were TNBC. Right-sided tumors were (*n* = 153, 48.9%) and left-sided were (*n* = 160, 51.1%). Multifocality was found in 61 (19.5%). In our data, patients most commonly presented with T4 (*n* = 140, 44.7%) and N1 stages (*n* = 131, 41.9%). Patient's age, side, and focality of tumor did not show any significant correlation with molecular classification. Grade III was more frequently noted in TNBC (*n* = 37, 48.1%) vs (*n* = 49, 20.8%) in non-TNBC group.

### 3.2. Ultrasound Findings and Luminal Classification (Table 3)

Ultrasound findings were normal in 2 (0.6%) patients, both belonging to Her-2 enriched category, which on mammogram showed only microcalcifications. The remaining 311 (99.4%) patients presented as a mass with or without calcifications. Majority of these tumors presented with irregular/spiculated margins (*n* = 274, 87.5%) (*p*=0.01). Hormone receptor (HR) positive and Her-2+ve tumors share almost similar distribution for spiculated margins, i.e., (*n* = 58, 93.5%), (*n* = 70, 98.6%), (*n* = 45, 95.7%), and (*n* = 52, 92.9%) for luminal A, B-1, B-2, and Her-2 +ve, respectively. Although most TNBC also presented with irregular margins (*n* = 36, 46.8%) but among tumors with well-circumscribed (*n* = 15, 4.8%) or microlobulated (*n* = 44, 14.1%) margins, TNBC showed significant correlation (*n* = 12, 15.6%) and (*n* = 29, 37.7%) (*p*=<0.01), respectively. Similarly, round- (*n* = 5, 1.6%) and oval-shaped (*n* = 32, 10.2) masses correlated well with TNBC (*p*=<0.01). Regardless of subtype of breast cancer, most of the tumors were hypoechoic (*n* = 263, 84%) (*p*=0.03). Only 36 (11.5%) masses appeared complex on sonography.

Triple-negative breast cancer (TNBC) exhibited a significant association with posterior acoustic enhancement. Among the tumors that were showing posterior acoustic enhancement, only almost half (*n* = 39, 50.6%) (*p*=<0.01) were TNBC. Majority tumors with posterior acoustic shadowing only (*n* = 131, 41.9%) showed HR positivity (*n* = 34, 54.8%) for luminal A and (*n* = 31, 43.7%) and (*n* = 16, 34.0%) for luminal B-1 and B-2 (*p* = 0.02). The majority of Her-2 enriched tumors also displayed only posterior shadowing (*n* = 29, 51.8%). In (*n* = 32, 10.2%), both features were present and were most commonly seen in B-1 tumors (*n* = 12, 16.9%) (*p*=0.01). In (*n* = 73, 23.3%) (*p*=<0.01), both features were absent.

In our study, the majority of tumors (*n* = 202, 64.55%) showed peripheral echogenic halo, among which the most common was luminal A (*n* = 56, 90.3%) (*p*=<0.01). With regard to orientation, the preponderance of malignant masses showed antiparallel orientation to the chest wall (*n* = 297, 94.9%) (*p*=0.03).

### 3.3. Mammography Findings and Luminal Classification (Table 4)

Mammography findings were normal in 7 (2.2%) patients and (*n* = 7, 2.2%) patients with focal asymmetry. In all these patients, ultrasound showed noncalcified mass. The most common mammographic feature was the presence of a mass without microcalcification (*n* = 197, 62.9%) followed by a mass with microcalcification (*n* = 92, 29.4%). Most common noncalcified tumors showed correlation with HR negativity (*n* = 64, 83.1%) (*p*=<0.01) followed by luminal A (*n* = 45, 72.6%). In contrast, Her-2 positive tumors (Her-2 enrich and luminal B-2) were significantly associated with microcalcifications, with (*n* = 29, 51.8%) or without associated mass (*n* = 29, 51.8%) (*p*=<0.01).

### 3.4. Multivariate Analysis (Table 5)

We also performed an analysis on combined imaging findings. Breast malignancies with round/oval shape and well-circumscribed/microlobulated margins were strongly associated with the absence of tumor markers—TNBC (*p*=<0.01) (O. R = 2.612) (CI = 6.034–30.744). Combining the presence of posterior acoustic enhancement and the absence of posterior acoustic shadowing with round/oval shape and well-circumscribed/microlobulated margins increased significantly the probability of the tumor being hormone receptor negative (*p*=0.01) (OR = 2.946) (CI = 8.224–44.073), (*n* = 24, 31.2%). Multivariate analysis for grades and Ki-67 showed significant association of TNBC with high-grade tumors (*p* < 0.01) (OR = 1.231) (CI = 1.962–5.977) and high Ki-67 (>14%) (*p*=0.03) (O. R = 1.108) (CI = 1.451–6.320). Presence of microcalcification with or without mass showed significant relationship with Her-2 positive tumors (*p*=<0.01) (O. R = 1.366) (CI = 2.371–6.481). Combining irregular margins and shape with the presence of microcalcifications with or without mass also displayed a significant relationship with Her-2 positivity (*p*=<0.01) (OR = 1.278) (CI = 2.145–6.004).

## 4. Discussion

We studied the correlation of sonographic and digital mammographic features with molecular classification of breast cancer and also devised a score called sono-mammometry score to anticipate the presence of non-TNBC tumors. We also determined the association of baseline histopathological type, grade of tumor, Ki-67, and T and N stages of the breast cancers with each luminal classification. We used the term “Her-2 enrich” for tumors that are hormone receptor negative and Her-2 neu +ve, while the term “Her-2 +ve” is used to indicate both luminal B-2 and Her-2 enrich tumors collectively.

We studied that non-triple-negative breast cancers are predominantly hypoechoic, less commonly complex in the echo pattern. These tumors strongly correlate with the presence of irregular spiculated margins along with peripheral echogenic halo and presence of posterior acoustic shadowing. Architectural distortion and trabecular thickening are also found frequently in this same subset. Presence of microcalcifications also correlates significantly with non-TNBC.

Spiculations in the margin are established criteria in the diagnosis of malignancy. Spiculated margins are generally secondary to drawing in of Cooper's ligaments into a tumor mass or the invasion of tumor cells into the neighboring breast tissues [11]. These irregular margins are believed to be associated with slow multiplication of tumor cells, which gives enough time for stromal interactions and induces fibrosis surrounding the invasive edge [11, 12]. Liu et al. [11] reported that masses with spiculated margins were significantly more common in patients with luminal A breast cancer than in those with other subtypes; however, we studied that HR-positive tumors (all non-TNBC) including Her-2 +ve luminal B and Her-2 enrich cancers presented with predominantly spiculated margins and we also deduced that because of this same reason non-TNBC tumor more often causes architectural distortion/trabecular thickening as secondary sign of malignancy. In our study, multivariate logistic regression analysis also determined the convincing association of irregular spiculated margins with Her-2 positivity. This is opposed to Liu et al.'s study [11] which determined that Her-2 overexpressing cancer almost never presents as spiculated masses. On the contrary, this is similar to the studies done by Lee et al. [3], Cho [4], and Elias et al. [13] who stated that circumscribed margins decreased the chance of HER-2 overexpression.

Lacroix et al. [14] determined that grade 1 and grade 2 tumors result in spicules and perilesional hyperechogenic halo. Our study supports this finding because, as discussed above, irregular margins were frequently associated with non-TNBC and this is the same subgroup that is more often related to low-grade (grade I and II) tumors. We also assume that peripheral echogenic halo which dictates the presence of desmoplastic reaction [10] can also be found routinely in this same luminal A, B and Her-2 enriched subgroups.

**Figure 2:**
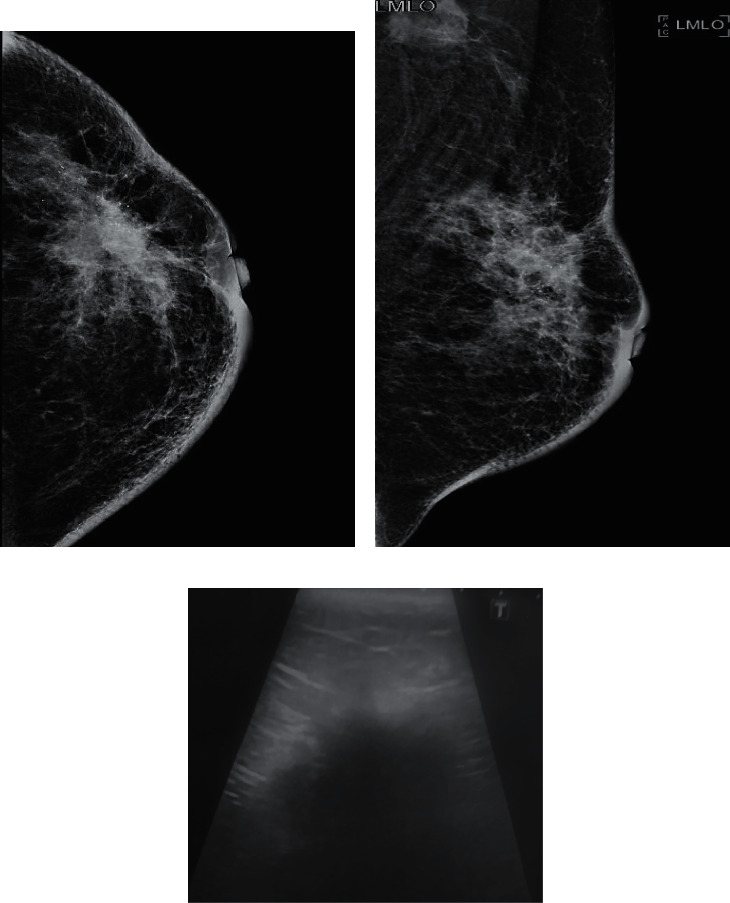
CC and MLO views of left breast: biopsy-proven hormone receptor +ve tumor (grade II). (a) A high-density mass is seen in upper-outer quadrant extending to retroareolar region. Irregular spiculated margins are seen with secondary signs of architectural distortion and trabecular thickening. (b) Overlying skin shows thickening and dimpling. Clustered microcalcifications are also seen within the mass. We can also see large malignant looking nodal mass in ipsilateral axilla. (c) Ultrasound of the same patient: there is a hypoechoic mass with irregular spiculated margins. Dense posterior acoustic shadowing is also seen obscuring posterior extent of the mass. We can also appreciate antiparallel orientation and peripheral echogenic halo. Sono-mammometry score = 14.

In invasive carcinomas, the unregulated and disorganized growth of malignant cells generates different tissue layers with variable acoustic impedance, which in turn causes an increase in the attenuation of the sound beams. This, along with fibrosis secondary to stromal interaction, results in posterior acoustic shadowing in sonographic images [10, 12]. We found a significant correlation of both HR-positive and Her-2 enriched tumors with the presence of posterior acoustic shadowing (Figures 2(a)–2(c)).

**Figure 3:**
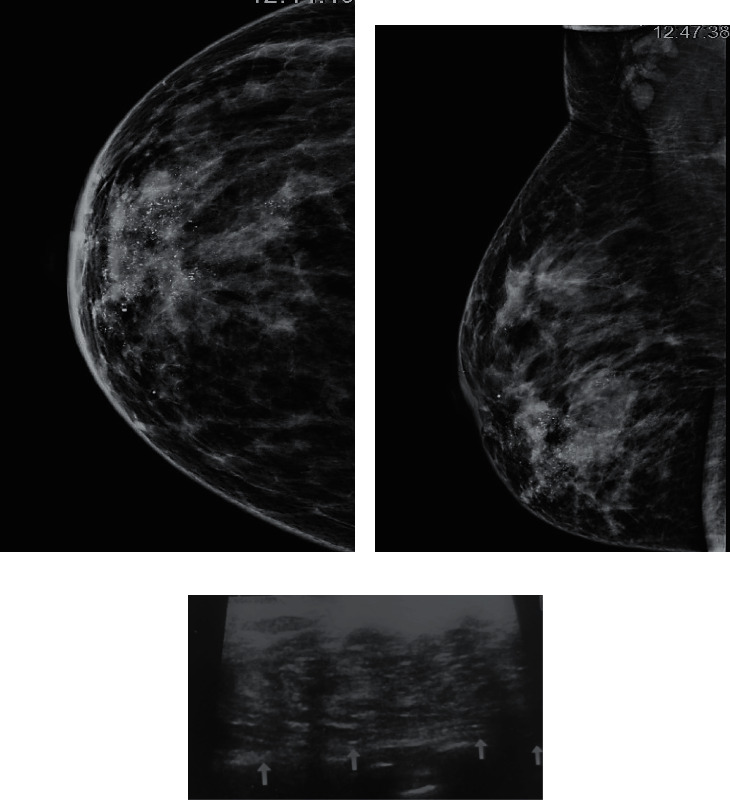
CC and MLO views of right breast. (a) Biopsy-proven infiltrating ductal carcinoma Her-2 enrich tumor (grade I). There is a high-density mass in retroareolar region extending to the lower quadrant. Irregular spiculated margins are seen along with visualization of extensive microcalcification within the mass. (b) Overlying skin thickening is also seen. Suspicious axillary lymph nodes are also there. (c) Ultrasound of the same patient shows a complex mass with partly ill-defined and partly irregular anterior margins. Abrupt boundary is seen with parallel orientation of this mass with the chest wall (wider than taller). No posterior features. Sono-mammometry score: 11.

**Figure 4:**
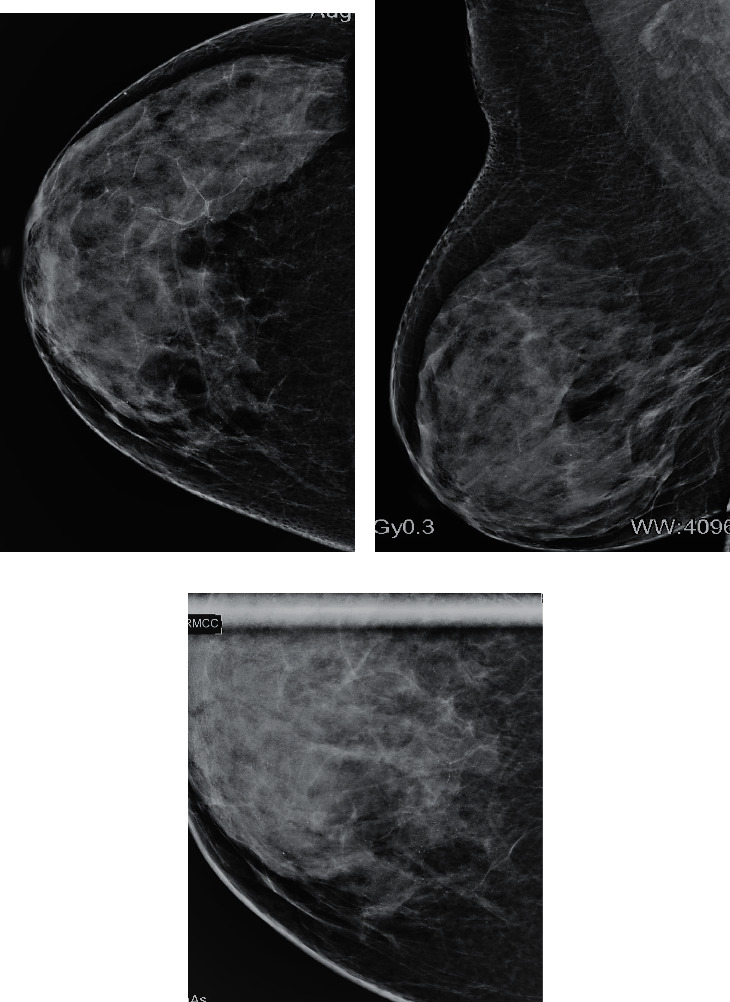
CC, MLO, and magnified CC view of right breast. (a) Biopsy-proven infiltrating ductal carcinoma Her-2 neu enrich tumor. (b) There are suspicious microcalcifications in the lower-outer quadrant which lead us to do a magnified CC view which shows clustered microcalcifications. (c) No definite mass was identified on mammogram as well as on ultrasound. Sono-mammometry score = 0.

It is imperative that the presence of microcalcifications at mammography is not definite for any breast cancer subtype [3]. These are more likely encountered in Her-2 +ve and HR-positive tumors [3]. In our study, we have divided luminal B cancers into two subgroups to scrutinize the impact of Her-2 positive status on imaging features. Multivariate logistic regression analysis demonstrates a convincing interconnection of microcalcifications (with or without the presence of a malignant mass) with Her-2 +ve disease. Cen et al. [15] stated that Her-2 enriched tumors were more expected to have heterogeneous and pleomorphic microcalcifications on mammogram. Likewise, Elias et al. [13] and Lee et al. [16] found that fine, pleomorphic or fine linear branching calcifications are the most characteristics findings for Her-2 +ve cancers. It is noteworthy that Her-2 +ve tumors belong to the subgroup that can be missed with ultrasound because of the presence of microcalcifications only [5]. In our study, two cases, in which ultrasound did not show any mass, had microcalcifications on mammogram and belong to Her-2 +ve subset (Figures 3(a)–3(c) and Figures 4(a)–4(c)).

**Figure 5:**
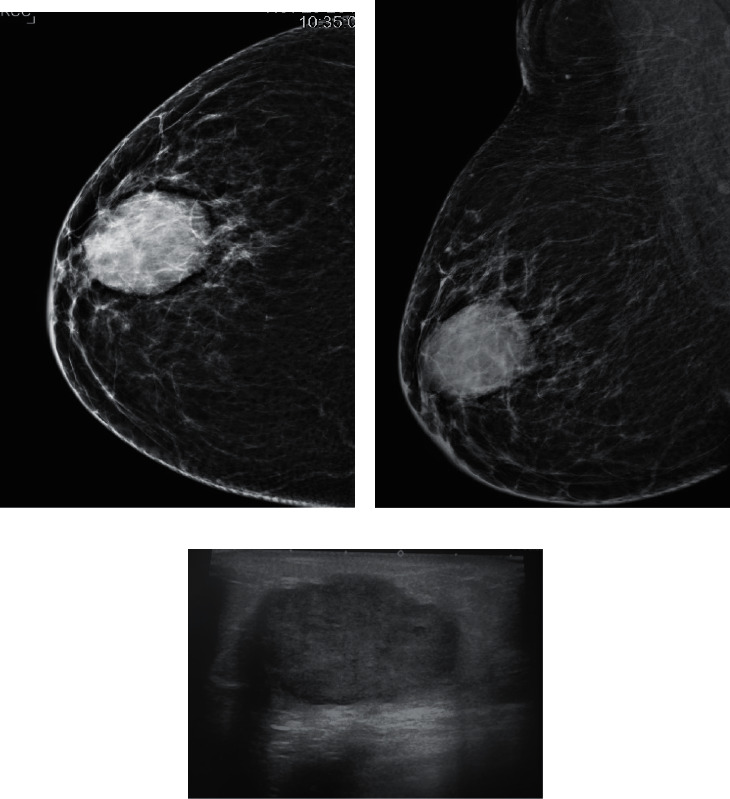
CC and MLO views of right breast. (a) Biopsy-proven metaplastic carcinoma triple-negative (grade III). There is an oval-shaped high-density mass in retroareolar region. Well-defined margins are seen. (b) Peripheral lucent halo is also seen. No microcalcifications are identified. (c) Ultrasound of the same patient shows a hypoechoic mass with microlobulated margins. Posterior acoustic enhancement is seen. Abrupt boundary with antiparallel orientation is also noticed. Sono-mammometry score = 9.

TNBC has been known to be associated with considerable differences in clinical, radiological, and pathological features compared with its counterpart [12, 17]. This is the subtype most discussed in the literature and it is the most commonly identified subgroup in our study. TNBC are also hypoechoic or show a complex echo pattern. We studied that TNBC can less commonly share imaging features similar to non-TNBC, i.e., mass with irregular margins and posterior shadowing. Imaging features that are distinctive to TNBC are similar to those that are also peculiar to benign tumors like noncalcified masses with well-circumscribed or microlobulated margins with posterior acoustic enhancement and more often with an abrupt boundary. Our findings were supported by studies by Lee et al. [3] and Lin et al. [12] who determined that benign “pseudofibroadenoma” type features can often be seen in TNBC. On sonography, the smooth well-circumscribed and microlobulated margin in TNBC is termed as “pushing border” microscopically and is considered secondary to rapid growth and high proliferation rate of malignant cells which leads to the lack of both stromal interaction and fibrosis. Cooper ligaments are believed to be displaced and not disrupted significantly in TNBC [10,12]. Identical to the benign masses, orderly and nestled growth of tumor cells in TNBC create fewer layers that lead to improved enhanced through transmission [10]. In addition, these tumors show a tendency towards being high grade and are more cellular and therefore display posterior acoustic enhancement more frequently than non-TNBC [12, 17]. Peripheral echogenic halo is believed to be less often found in TNBC. Our findings were also supported by studies done by Lacroix et al. [14] and Wojcinski et al. [10] who also found abrupt tumor parenchyma interface more frequently in these tumors. TNBC are also known to lack the presence of suspicious microcalcifications on mammogram [3, 18] (Figures 5(a)–5(c)). However, Lin et al. [12] believed that there are wide variations in imaging features for TNBC. In our study, we postulate that close resemblance of its imaging features with benign tumors warrants improving diagnostic performance for early suspicion and recognition of malignancy, particularly in outpatient clinics where prompt and precise decisions are required.

Although there are some characteristics imaging features that can be attributed to each luminal category, overall there is considerable overlap in imaging features of these 5 groups. Therefore, based on our results, we divided these cancers into two groups, non-TNBC and TNBC, and devised a score called “sono-mammometry” score (Table 5) consisting of simple sonological and mammographic features for prediction of non-TNBC and TNBC which can be easily performed in outpatient settings. Score of 10.5 shows sensitivity of 94%. Its low specificity of 47% is a limitation which warrants validation of this score on a larger number of patients before future implication. However, this score is still useful, particularly in a developing country where resources are limited. Although histopathological diagnosis providing us with indispensable information regarding tumor biology and histological interpretation cannot be ignored, all laboratory tests have the incidence of false results [11]. It highlights the importance of a radiologist to be well-aware of common and uncommon imaging features, so a question can be raised in the matter of conflicting lab results and perhaps it may be beneficial to repeat testing of the immunohistochemical receptors.

It is worthwhile to discuss the advancement of artificial intelligence with regard to medical imaging. In our study, human readers are involved in anticipating results with the help of simple features on mammogram and ultrasound; however, there are studies which have incorporated newer imaging techniques, e.g., Dilorenzo et al. [19] has shown the potential role of background parenchymal enhancement in MRI breast in discriminating different breast cancer subtypes. Fusion of ultrasound and MRI images also proved to provide information about additional occult breast lesions [20]. State-of-the-art techniques assimilate radiomics in discerning benign and malignant breast lesions on mammography [21, 22]. Different variety of machine learning techniques has also been developed and integrated for early detection of breast cancer through textural analysis of microcalcification on mammogram and for further classification of breast cancers [23–25]. Contrast-enhanced spectral mammography is a recently introduced mammographic method in which Forgia et al. have discussed imaging features of two cancer molecular subtypes, i.e., HER-2 positive and triple-negative, with the help of radiomics [26]. Although radiomics is an evolving methodology which has shown to provide insightful results in quantitatively and objectively elucidating tumor biology, there are issues of standardization and lack of reporting guidelines. Furthermore, in economically developing and resource-constrained environment, these advanced engineering techniques are still seemed to be far-fetched and human readers with manual interpretation of images take precedence.

Our results should be comprehended after taking into account the limitations. First, it is a retrospective study with a small number of patients. Prospective studies with larger numbers of patients are needed to validate these results. Sono-mammometry score should be applied in a prospective population reassessment to see how predictive the model obtained is. Another is that breast density was not taken into account. Moreover, our analysis was carried out on selected images rather than images acquired during real-time scanning and operator-dependent nature of ultrasound can give rise to biases.

## 5. Conclusion

Breast cancer exhibits different imaging features according to molecular type. Although imaging characteristics are helpful in the prediction of molecular classification, the prognostication of these features is still weak. There is considerable variation in the imaging features of TNBC as some of its imaging features overlap with those of typical benign tumors, which warrants vigilance towards improved diagnostic performance for early suspicion and recognition of malignancy. To help better understand these features, our sono-mammometry score can serve as a relatively inexpensive and straightforward test which assumes to be functional and productive in resource-limited settings. Our next step would be to apply this score in prospective study to assess its predictive value.

## Figures and Tables

**Table 1:** Sono-mammometry score.

Imaging findings			
Only pleomorphic microcalcifications (without mass or focal asymmetry on mammogram or ultrasound) (0)			
Mass/focal asymmetry	Without microcalcification (+1)	With microcalcification (+2)	—
Mass shape	Round (+1)	Oval (+2)	Irregular, spiculated (+3)
Mass margins	Well circumscribed (+1)	Microlobulated (+2)	Irregular-spiculated/ill-defined/obscured (+3)
Mass boundary	Abrupt (+1)	Echogenic halo (+2)	—
Posterior shadowing	Absent (+1)	Present (+2)	—
Posterior enhancement	Absent (+1)	Present (+1)	—
Orientation	Parallel (+0)	Antiparallel (+1)	—

Max. score: 14			

**Table 2:** Demographic and histopathological characteristics.

Clinicoradiological features of breast cancers	Total (*n* = 313) (%)	Luminal A (*n* = 62)	Luminal B1 (Her-2-ve) (*n* = 71)	Luminal B2 (Her-2+ve) (*n* = 47)	TNBC (*n* = 77)	Her-2–neu enrich (*n* = 56)	*p* value
Age							
20–30	16 (5.1)	1 (1.6)	2 (2.8)	2 (4.3)	7 (9.1)	4 (7.1)	0.3
30–50	165 (52.7)	33 (53.2)	35 (49.3)	30 (63.8)	41 (53.2)	26 (46.4)	
50–80	132 (42.2)	28 (45.2)	34 (47.9)	15 (31.9)	29 (37.7)	26 (46.4)	

Side							
Right	153 (48.9)	32 (51.6)	38 (53.5)	20 (42.6)	36 (46.8)	27 (48.2)	0.79
Left	160 (51.1)	30 (48.4)	33 (46.5)	27 (57.4)	41 (53.2)	29 (51.8)	

Mammography findings							
Negative	7 (2.2)	0 (0.0)	2 (2.8)	3 (6.4)	1 (1.3)	1 (1.8)	0.232
Positive	306 (97.8)	62 (100.0)	69 (97.2)	44 (93.6)	76 (98.7)	55 (98.2)	

Ultrasound findings							
Negative	2(0.6)	0(0.0)	0(0.0)	0(0.0)	0 (0.0)	2 (3.6)	0.05
Positive	311(99.4)	62(100.0)	71(100.0)	47(100.0)	77 (100.0)	54 (96.4)	

Focality							
Unifocal	252 (80.5)	48 (77.4)	53 (74.6)	37 (78.7)	65 (84.4)	49 (87.5)	0.34
Multifocal	61 (19.5)	14 (22.6)	18 (25.4)	10 (21.3)	12 (15.6)	7 (12.5)	

T stage							
T0	2 (0.6)	0 (0.0)	0 (0.0)	0 (0.0)	0 (0.0)	2 (3.6)	0.06
T1	30 (9.6)	3 (4.8)	8 (11.3)	9 (19.1)	4 (5.2)	6 (10.7)	
T2	112 (35.8)	26 (41.9)	31 (43.7)	15 (31.9)	27 (35.1)	13 (23.2)	
T3	29 (9.3)	6 (9.7)	5 (7.0)	3 (6.4)	10 (13.0)	5 (8.9)	
T4	140 (44.7)	27 (43.5)	27 (38.0)	20 (42.6)	36 (46.8)	30 (53.6)	

N stage							
N0	93 (29.7)	16 (25.8)	23 (32.4)	11 (23.4)	26 (33.8)	17 (30.4)	0.81
N1	131 (41.9)	27 (43.5)	28 (39.4)	22 (46.8)	34 (44.2)	20 (35.7)	
N2	89 (28.4)	19 (30.6)	20 (28.2)	14 (29.8)	17 (22.1)	19 (33.9)	

Histopathology							
IDC^*∗*^	273 (87.2)	47 (75.8)	62 (87.3)	44 (93.6)	66 (85.7)	54 (96.4)	0.01
ILC^*∗*^	22 (7.0)	7 (11.3)	7 (9.9)	3 (6.4)	4 (5.2)	1 (1.8)	
Metaplastic carcinoma	10 (3.2)	2 (3.2)	0 (0.0)	0 (0.0)	7 (9.1)	1 (1.8)	
Mucinous carcinoma	4 (1.3)	3 (4.8)	1 (1.4)	0 (0.0)	0 (0.0)	0 (0.0)	
Invasive papillary carcinoma	3 (1.0)	2 (3.2)	1 (1.4)	0 (0.0)	0 (0.0)	0 (0.0)	
Invasive carcinoma with neuroendocrine features	1 (0.3)	1 (1.6)	0 (0.0)	0 (0.0)	0 (0.0)	0 (0.0)	

Grade							
I	34 (10.9)	14 (22.6)	9 (12.7)	5 (10.6)	2 (2.6)	4 (7.1)	<0.01
II	193(61.7)	36 (58.1)	48 (67.6)	28 (59.6)	38 (49.4)	43 (76.8)	
III	86 (27.5)	12 (19.4)	14 (19.7)	14 (29.8)	37 (48.1)	9 (16.1)	

^*∗*^ IDC: infiltrating ductal carcinoma, ^*∗*^ ILC: Infiltrating lobular carcinoma, ^*∗*^ TNBC: triple-negative breast cancer.

**Table 3:** Ultrasound findings.

Imaging characteristics	Total (*n* = 313)	Luminal A (*n* = 62)	Luminal B1 (*n* = 71)	Luminal B2 (*n* = 47)	TNBC (*n* = 77)	Her-2 enrich (*n* = 56)	*p* value
Imaging findings							
Negative	2 (0.6)	0 (0.0)	0 (0.0)	0 (0.0)	0 (0.0)	2 (3.6)	0.05
Positive	311 (99.4)	62 (100)	71 (100)	47 (100)	77 (100)	54 (96.4)	

Mass (*n* = 311)							
Shape							
Round	5 (1.6)	0 (0)	0 (0)	1 (2.1)	4 (5.2)	0 (0)	<0.01
Oval	32 (10.2)	4 (6.5)	1 (1.4)	1 (2.1)	24 (31.2)	2 (3.6)	
Irregular	274 (87.5)	58 (93.5)	70 (98.6)	45 (95.7)	49 (63.6)	52 (92.9)	

Echogenicity							
Hypoechoic	263 (84)	51 (82.3)	65 (91.5)	42 (89.4)	59 (76.6)	46 (82.1)	0.03
Hyperechoic	1 (0.3)	0 (0)	0 (0)	1 (2.1)	0 (0)	0 (0)	
Isoechoic	11 (3.5)	4 (6.5)	1 (1.4)	1 (1.4)	2 (2.6)	2 (3.6)	
Complex	36 (11.5)	7 (11.3)	5 (1.6)	2 (4.3)	16 (5.1)	6 (10.7)	

Margins							
*Well circumscribed*	15 (4.8)	1 (1.6)	0 (0)	0(0)	12(15.6)	2(3.6)	<0.01
*Non-well circumscribed*	—	—	—	—	—	—	
Microlobulated	44 (14.1)	5 (8.1)	2 (2.8)	7 (14.9)	29 (37.7)	1 (1.8)	
Irregular/spiculated	252 (80.5)	56 (90.3)	69 (97.2)	40 (85.1)	36 (46.8)	51 (91.1)	

Orientation							
Parallel	14 (4.5)	4 (6.5)	1 (1.4)	1 (0.3)	7 (2.2)	1 (1.8)	0.03
Antiparallel	297 (94.9)	58 (93.5)	70 (98.6)	46 (97.9)	70 (90.9)	53(94.6)	

Posterior features							
Enhancement only	75 (24)	7 (11.3)	6 (8.5)	3 (6.4)	39 (50.6)	20 (35.7)	<0.01
Shadowing only	131 (41.9)	34 (54.8)	31 (43.7)	16 (34.0)	21 (27.3)	29 (51.8)	0.02
Both present	32 (10.2)	5 (8.1)	12 (16.9)	8 (17)	3 (3.9)	4 (7.1)	0.01
Both absent	73(23.3)	16 (25.8)	22 (31.0)	20 (42.6)	14 (18.2)	1 (1.8)	<0.01

Boundary							
Abrupt	109(34.8)	6 (9.7)	15 (21.1)	26 (55.3)	41 (53.2)	21 (37.5)	<0.01
Echogenic halo	202(64.5)	56 (90.3)	56 (78.9)	21 (44.7)	36 (46.8)	33 (58.9)	

^*∗*^TNBC: triple-negative breast cancer.

**Table 4:** Mammographic findings.

Imaging characteristics	Total (*n* = 313)	Luminal A (*n* = 62)	Luminal B-1 (Her-2−ve) (*n* = 71)	Luminal B-2 (Her-2+ve) (*n* = 47)	TNBC (*n* = 77)	Her-2 enrich (*n* = 56)	*p* value
Findings							
Negative	7 (2.2)	0 (0.0)	2 (2.8)	3 (6.4)	1 (1.3)	1 (1.8)	<0.01
Mass only	197 (62.9)	45 (72.6)	46 (64.8)	22 (46.8)	64 (83.1)	20 (35.7)	
Microcalcifications only	10 (3.2)	1 (1.6)	1 (1.4)	4 (8.5)	0 (0.0)	4 (7.1)	
Mass with microcalcifications	92 (29.4)	15 (24.2)	19 (26.8)	17 (36.2)	12 (15.6)	29 (51.8)	
Focal asymmetry	7 (2.2)	1 (1.6)	3 (4.2)	1 (2.1)	0 (0.0)	2 (3.6)	

Mass (*n* = 289)							
Margins							<0.01
*Well circumscribed*	15 (4.8)	1 (1.6)	0 (0.0)	0 (0.0)	12 (15.6)	2 (3.6)	
*Non-well circumscribed*	—	—	—	—	—	—	
Microlobulated	40 (12.8)	5 (8.1)	1 (1.4)	5 (10.6)	28 (36.4)	1 (1.8)	
Spiculated/obscured/indistinct	234 (74.8)	54 (87.1)	64 (90.1)	34 (72.3)	36 (46.8)	46 (82.1)	

Architectural distortion/trabecular thickening							
Yes	226 (72.2)	46 (74.2)	59 (83.1)	34 (72.3)	39 (50.6)	48 (85.7)	<0.01
No	87 (27.8)	16 (25.8)	12 (16.9)	13 (27.7)	38 (49.4)	8 (14.3)	

^*∗*^TNBC: triple-negative breast cancer.

**Table 5:** Multivariate analysis of ultrasound and mammographic findings.

Imaging findings	Outcome variable	*p* value	Odds ratio	Confidence interval
Noncalcified mass/focal asymmetry v/s mass with calcification/only microcalcifications	TNBC^*∗*^ v/s non-TNBC^*∗*^	0.03	1.124	6.141–22.69
Mass shape round/oval v/s irregular	TNBC v/s non-TNBC	<0.01	2.443	4.836–27.382
Margins well-circumscribed/microlobulated v/s irregular spiculated	TNBC v/s non-TNBC	<0.01	2.472	1.462–6.479
Orientation parallel v/s nonparallel	TNBC v/s non-TNBC	0.03	1.176	1.100–9.562
Posterior enhancement v/s shadowing	TNBC v/s non-TNBC	<0.01	1.731	3.190–9.988
Round/oval shape + well-defined/microlobulated margins	TNBC v/s non-TNBC	<0.01	2.612	6.034–30.744
Well-circumscribed/microlobulated margins + presence of posterior enhancement + absence of posterior shadowing	TNBC v/s non-TNBC	<0.01	2.946	8.224–44.073
Tumor grade III v/s I/II	TNBC v/s Non-TNBC	<0.01	1.231	1.962–5.977
Ki-67 high (<14%) v/s low (<14%)	TNBC v/s non-TNBC	0.03	1.108	1.451–6.320
Mass with microcalcifications/only microcalcifications v/s noncalcified mass/focal asymmetry	Her-2 neu +ve v/s Her-2 neu –ve	<0.01	1.366	2.371–6.481
Irregular shape/margins v/s well-circumscribed margins	Her-2 neu +ve v/s Her-2 neu –ve	.009	.937	1.263–5.157
Mass with microcalcifications/only microcalcification + irregular margins	Her-2 neu +ve v/s Her-2 neu –ve	<0.01	1.278	2.145–6.004

^*∗*^TNBC: triple-negative breast cancer. ^*∗*^Non-TNBC:non-triple-negative breast cancer.

## Data Availability

The data used in the study are available from the corresponding author (Sana Shaikh) upon request.
